# Differential activation of amygdala, dorsal and ventral hippocampus following an exposure to a reminder of underwater trauma

**DOI:** 10.3389/fnbeh.2014.00018

**Published:** 2014-01-29

**Authors:** Gilad Ritov, Ziv Ardi, Gal Richter-Levin

**Affiliations:** ^1^Sagol Department of Neurobiology, University of HaifaHaifa, Israel; ^2^The Institute for the Study of Affective Neuroscience (ISAN), University of HaifaHaifa, Israel; ^3^Psychology Department, University of HaifaHaifa, Israel

**Keywords:** ventral hippocampus, amygdala, emotional memory, PTSD, rat model

## Abstract

Recollection of emotional memories is attributed in part to the activation of the amygdala and the hippocampus. Recent hypothesis suggests a pivotal role for the ventral hippocampus (VH) in traumatic stress processing and emotional memory retrieval. Persistent re-experiencing and intrusive recollections are core symptoms in acute and posttraumatic stress disorders (ASD; PTSD). Such intrusive recollections are often triggered by reminders associated with the trauma. We examined the impact of exposure to a trauma reminder (under water trauma (UWT)) on the activation of the basolateral amygdala (BLA), dorsal and VH. Rats were exposed to UWT and 24 h later were re-exposed to the context of the trauma. Phosphorylation of the extracellular signal-regulated kinase (ERK) was used as a marker for level of activation of these regions. Significant increase in ERK activation was found in the VH and BLA. Such pattern of activation was not found in animals exposed only to the trauma or in animals exposed only to the trauma reminder. Additionally, the dissociative pattern of activation of the VH sub-regions positively correlated with the activation of the BLA. Our findings suggest a specific pattern of neural activation during recollection of a trauma reminder, with a unique contribution of the VH. Measured 24 h after the exposure to the traumatic experience, the current findings relate to relatively early stages of traumatic memory consolidation. Understanding the neural mechanisms underlying these initial stages may contribute to developing intervention strategies that could reduce the risk of eventually developing PTSD.

## Introduction

Traumatic memories re-experiencing is a core symptom in the diagnosis of acute stress disorder (ASD) and posttraumatic stress disorder (PTSD). Re-experiencing, which includes elements of recurrent and intrusive recollections, may be triggered by reminders associated with the traumatic event (American Psychiatric Association, 2013). This phenomenon has been conceptualized in terms of conditioned fear responses, elicited by enhanced emotional memory mediated by a hyper-responsive amygdala (Gilboa et al., 2004). Correspondingly, both human and animal studies have shown that the amygdaloid complex is crucial for encoding and retrieval of conditioned fearful memories (Armony and LeDoux, 1997; Liberzon et al., 1999) and that the basolateral amygdala (BLA) is essential for the retrieval of traumatic memories after a presentation of a reminder for these events (Tronel and Alberini, 2007). The amygdaloid complex is reciprocally connected with the hippocampus, mainly through the BLA and posterior cortical nuclei (Pitkanen et al., 2000). The hippocampus mediates declarative memory functions and plays an important role in the integration of memory elements at the time of retrieval by assigning significance for events within space and time (Squire and Zola-Morgan, 1991). It has been suggested that during an emotional experience the amygdala interprets the emotional value of the incoming information while attaching emotional significance to its different aspects (Richter-Levin and Akirav, 2003). This evaluation is then past to the hippocampus that forms a specific context for the events’ episodic memory. Thus, intensity of the incoming input from the amygdala correlates with the intensity of memory encoding in the hippocampus (Canli et al., 2000).

The functional connections between the hippocampus and amygdala seem to be centralized to the ventral parts of the hippocampus and the BLA of the amygdala (Pitkanen et al., 2000). In accordance, gene expression in the dorsal hippocampus (DH) was shown to correlate with cortical regions involved in information processing, while genes expression in the ventral hippocampus (VH) correlate with regions involved in emotion and stress such as the amygdala. These findings had led to the suggestion that the DH performs primarily cognitive functions while the VH relates to stress, emotion, and affect (for review see Fanselow and Dong, 2010).

In regard to stress response, a recent hypothesis by Segal et al. (2010) suggested that stress induces dynamic routing of hippocampal connectivity. According to this hypothesis, under normal state the hippocampus is linked to the rest of the brain primarily via its dorsal efferents, connected primarily with paleo- and neocortical areas. However, during a stressful experience the hippocampal functioning is modified such that it emphasizes more its ventral pole, and synchronizes its activity with other stress-related areas of the brain, such as the amygdala. This hypothesis is partially supported by gene expression studies of the DH and VH in rodents (Caudal et al., 2010; Fanselow and Dong, 2010) and imaging studies of anterior and posterior hippocampus during stress processing in humans (Satpute et al., 2012). However, there is no current support to this hypothesis in regard to VH and amygdala co-activation involvement in the retrieval of stressful memory.

Extracellular signal-regulated kinase (ERK) is suggested to represent an essential component of the signal transduction mechanisms sub-serving memory formation. Its activation was found to be required for the expression of long-term memory (LTM) induced by fear-conditioning paradigms and spatial learning in the hippocampus and amygdala (Adams and Sweatt, 2002; Sweatt, 2004). The ERK1/2 pathway was demonstrated to be activated in neuronal circuits of the hippocampus and amygdala following the retrieval of a contextual fear conditioning memory (Antoine et al., 2013). In accordance with that, studies in our lab have shown that exposure to a contextual reminder of a stressful experience was accompanied by ERK2 activation in the BLA of exposed rats (Ilin and Richter-Levin, 2009; Ardi et al., 2013).

In order to examine the predictions of the “dynamic routing hypothesis” (Segal et al., 2010) during the retrieval processes of contextual fear, we measured the phosphorylation of ERK in DH, VH and the BLA following re-exposure to the context of the “underwater trauma” (UWT), a paradigm designed to model sudden and brief traumatizing experiences (Richter-Levin, 1998; Wang et al., 2000; Cohen et al., 2004). We hypothesized that rats exposed to the combination of a contextual reminder, 24 h following an exposure to UWT, will show higher levels of ERK activation in the ventral regions of the hippocampus and in the BLA compared to rats exposed to the UWT or the reminder alone. Furthermore, we hypothesized that there will be a positive correlation between ERK activation in the BLA and ERK activation in the VH, but not DH, of rats that will be exposed both to the trauma and the trauma reminder.

## Materials and methods

### Participants

This study used *n* = 60 male Sprague Dawley (SD) rats weighing 200–224 g upon arrival (Harlan, Jerusalem, Israel).

Animals were housed in groups of three per cage, in a 35 × 60 × 18 cm Plexiglas cages in temperature-controlled (23°C +/− 1°C) animal quarters on a 12:12 h light-dark cycle (lights on 0700–1900 h). They had ad libitum access to standard rodent chow pellets and water.

### Swim experience

Rats were placed in a plastic tank (diameter 50 cm, height 60 cm) containing water (22 ± 2°C) 30 cm deep, for 1 min each day. This procedure lasted 5 consecutive days.

### Underwater trauma (UWT) stress

The UWT stress was carried out in the plastic tank that was used for the 5 days of swim. Rats were placed in the water and given 30 s of swim and then were held under water for additional 30 s using a special metal net (20 ×10 ×15 cm).

### Reminder

The exposure to the reminder was conducted 24 h after the exposure of the UWT rats’ to the underwater stress; and for “Swim” rats, 24 h after the exposure to the additional day of swim experience. Rats were placed back in the plastic tank (the same one that was used in the 5 days of swim experience and in the UWT stress) and were given a 30 s swim session. Following a 2 min period of drying in a neutral cage with dry sawdust rats were returned to their home cage until time for decapitation. Rats’ behavior in the water during the reminder was recorded on video for later analysis.

### Experimental design

The experiment was conducted through four sequential sets of trail runs. Each set comprised of *n* = 15 animals at a time, divided into five different cages (three animals per cage). Following a 3 days acclimation period, cages were randomly assigned to one of the experimental groups (UWT, Swim, and Naïve). UWT and Swim rats were exposed to 5 consecutive days of swim experience. On the 6*th* day, UWT rats were exposed to the UWT stress while Swim rats were exposed to additional day of swim experience. On the 7*th* day, half of rats from both UWT and Swim groups were exposed to the reminder while the rest of the rats remained in their home cage. Naïve rats were not exposed to the swim experience, UWT stress or to the reminder.

The study was approved by the ethics committee of Haifa University. Experiments were carried out in accordance with the Guidelines laid down by the NIH in the US regarding the care and use of animals for experimental procedures.

### Immunoblot analysis

**Figure 1:**
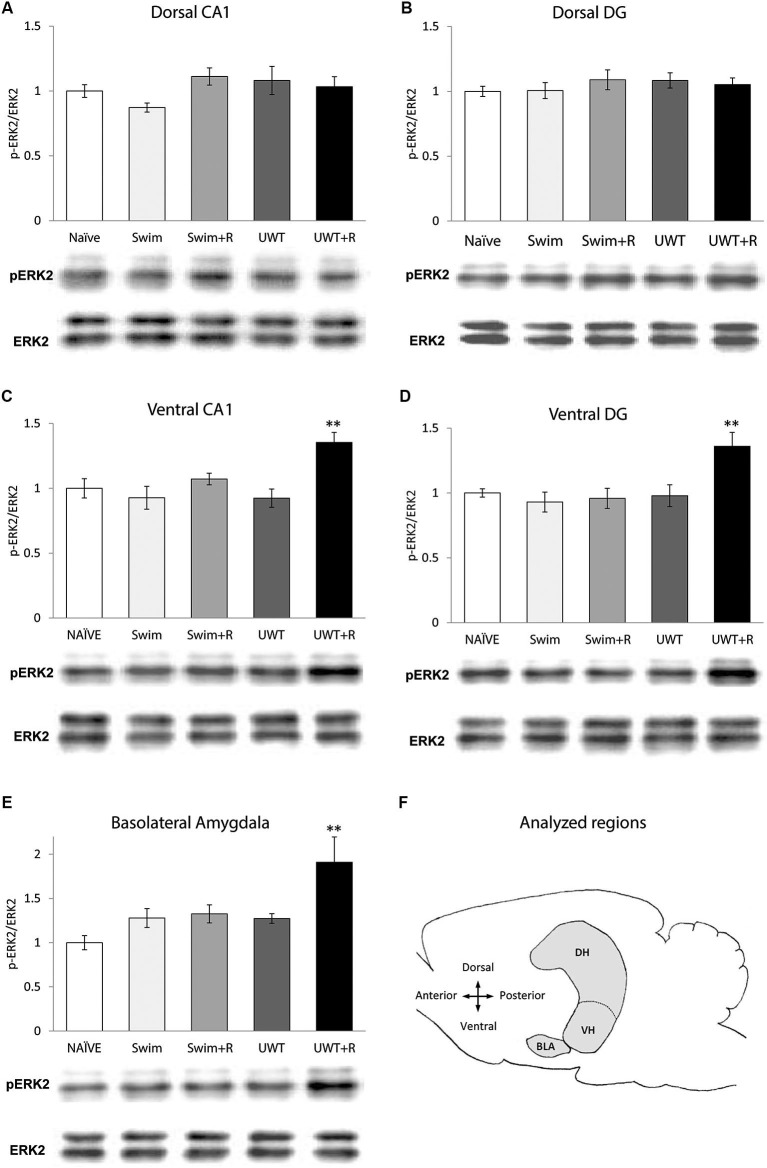
**Mean ± standard error of the mean (SEM) ERK2 activation 30 min after the exposure to reminder of a traumatic experience (*n* = 12 in each group). (A)** ERK2 activation in the dorsal CA1 did not differ significantly between the groups. **(B)** ERK2 activation in the dorsal DG did not differ significantly between the groups. **(C)** ERK2 activation in the ventral CA1 of the UWT + R group was significantly higher than the rest of the groups. **(D)** ERK2 activation in the ventral DG of the UWT + R group was significantly higher than the rest of the groups. **(E)** ERK2 activation in the BLA of the UWT + R group was significantly higher than the rest of the groups. **(F)** Diagram of analyzed regions. DH = dorsal hippocampus; VH = ventral hippocampus. ** *p* < 0.01.

Thirty minutes following the exposure to the reminder rats were decapitated, their brains were removed and semi-frozen by 1 min covering under dry ice powder. As depicted in Figure 1F, hippocampus dorsal and ventral regions (dorsal 4/5*th* of CA1, ventral 1/5*th* of CA1, dorsal 4/5*th* of Dentate Gyrus (DG) and ventral 1/5*th* of DG; In accordance to Segal et al. (2010) and BLA brain regions were incised bilaterally with a sterile 2 mm spatula according to the atlas of Paxinos and Watson (2005) (Anteroposterior coordinates: BLA: −1.60 to −2.80 from Bregma; DH: −2.80 to −6.00 from Bregma. Dorsoventral coordinates: 2.1 to 6.3 from Bregma; VH: −4.00 to −6.30 from Bregma. Dorsoventral coordinates: 6.3 to 9.2 from Bregma). The incised semi-frozen tissues were then collected into 1.5 ml Eppendorf tubes, immediately frozen in liquid nitrogen and stored at −80°C until further use. Tissues where homogenized in a glass Teflon homogenizer in 180–700 μl of ice-cold Urea Lysis Buffer (1 mM EDTA (Fluka), 0.5% Triton X (SIGMA), 6M Urea (SIGMA), 100 μM PMSF (SIGMA)) with freshly added protease and phosphotase inhibitors (0.1 mM sodium orthovanadate, 1 lg/ml leupeptine, 1.6 lg/ml aprotinin, 5 mM NaF, and 1 lg/ml protease inhibitor cocktail P2714 (from Sigma, St. Louis, MO)) and incubated at 100°C for 5 min. In accordance with Cohen-Matsliah et al. (2007), samples of 10 μg were loaded in two different lanes of the 10% SDS-polyacrylamide gel electrophoresis (SDS-PAGE). Following 1 h semi-dry transfer (60 mA per membrane) onto a 0.45 μm nitrocellulose membrane the lanes were compared for gross protein homogeneity loading by Ponceau staining (SIGMA) to identify and rerun blots with uneven lane loading. Blots were blocked using 3% BSA in Tris-Buffered Saline Tween-20 (TBST: 0.9% w/v NaCl, 0.05% v/v Tween-20 and 100 mM Tris- HCl, pH 7.6) incubation for 45 min at room temperature (RT). Membranes were then incubated in rabbit *α*-ERK1/2 (anti-p44/42 MAP Kinase (MAPK); 1:1000 in TBST; Cell Signaling Technology, Beverly, MA, USA) or *α*-p-ERK1/2 (phospho-p44/42 MAPK; Thr202/Tyr204; 1:1000 in TBST; Cell Signaling Technology, Beverly, MA, USA) overnight on a shaker at 4°C. The next day excess of first antibody was washed three times for 10 min with TBST. Secondary *α*-rabbit antibody incubation conducted for 1 h at RT. The membranes were washed three times, 10 min each, in TBST before development, with EZ-ECL chemiluminescence light reaction (Amersham, Piscataway, NJ) using the charge-coupled device (CCD) camera (XRS BioRad).

### Quantification

Densitometric analysis of ERK2 immunoreactivity was conducted in Quantity One 1-D Analysis software. Each sample was measured relative to the background, and phosphorylation levels were calculated as the optical density (OD) ratio between the phosphorylated (phospho-ERK2) and the nonphosphorylated (ERK2) forms of the protein. The results were normalized to the Naïve group values.**** Only exposures that were in the linear range of the ECL reaction were used for quantification analysis. Although the anti-phospho and anti-total ERK1/2 antibodies used in the present study recognized both ERK1 and ERK2, p-ERK1 was not quantified because the signals were often too faint and inconsistent to be accurately analyzed.

### Statistical analysis

Data are presented as the mean ± standard errors of the mean. One way ANOVA with LSD *post-hoc* and Pearson correlations were conducted using SPSS 15 software. Differences and correlations were considered statistically significant when *p* < 0.05.

## Results

### Behavior

**Figure 2:**
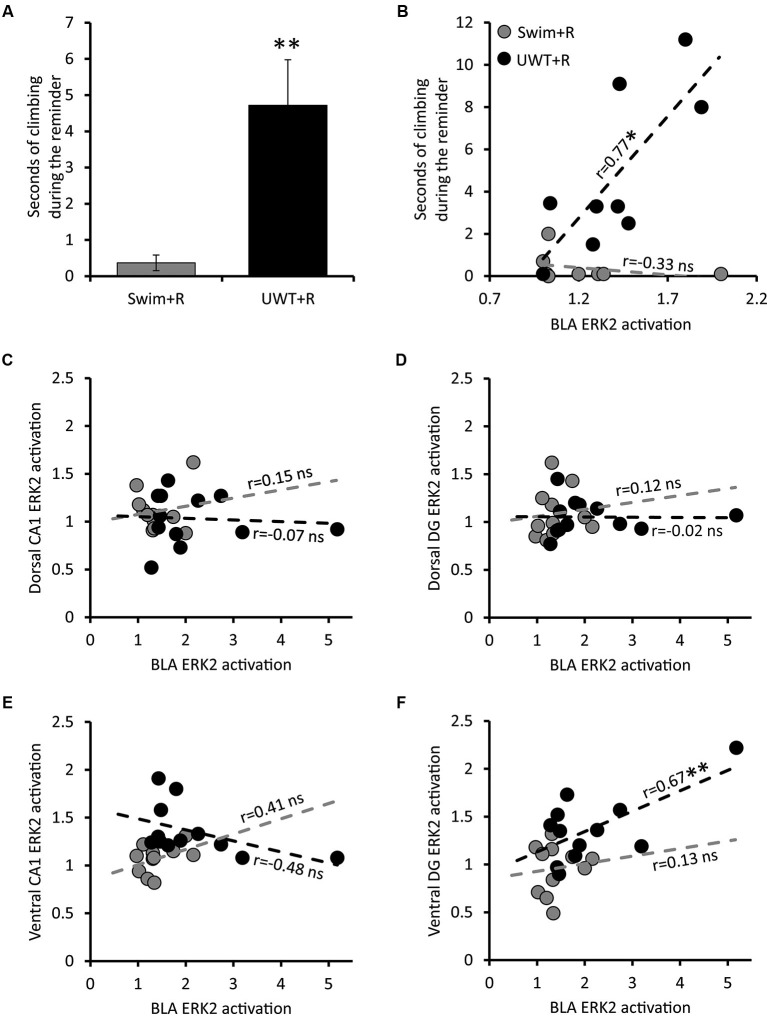
**Behavior and Pearson correlations of ERK2 activation of rats exposed to the reminder. (A)** Mean ± SEM of time spent wall climbing during the reminder (*n* = 9 in each group). **(B)** Correlation between the time spent wall climbing during the reminder and ERK2 activation in the BLA (*n* = 9 in each group). **(C)** Correlation between ERK2 activation in the BLA and ERK2 activation in the dorsal CA1 (*n* = 12 in each group). **(D)** Correlation between ERK2 activation in the BLA and ERK2 activation in the dorsal DG (*n* = 12 in each group). **(E)** Correlation between ERK2 activation in the BLA and ERK2 activation in the ventral CA1 (*n* = 12 in each group). **(F)** Correlation between ERK2 activation in the BLA and ERK2 activation in the ventral DG (*n* = 12 in each group). * *p* < 0.05; ** *p* < 0.01.

Twenty-four hours after the exposure of the UWT rats to the underwater stress and Swim rats to the additional day of swim experience, half of rats from each group (“UWT + R” and “Swim + R” hereafter) were placed back in the plastic tank for 30 s of swimming (i.e., Reminder). Due to a technical error, video recordings of the first set of the experiment were lost. Data of rats behavior therefore represent three sets of the experiment instead of 4 (Total *n* = 9 in each group). Analyses of rats’ behavior in the water revealed a unique pattern of swimming in the UWT + R group. This involved a frequent intermissions of swimming in which they attempted escaping by wall climbing, defined as upward directed movements of the forepaws along the side of the tank. This pattern was unique to the UWT + R group. Studies in the past suggest that this kind of behavior represents anxious attempts to escape (Cryan et al., 2005). To quantify this behavior, total wall climbing time was measured as an indication of the extent in which the reminder was stressful for the animal. Indeed, a significant difference in this measure was found between the two groups that were exposed to the reminder (*t*_(16)_ = 3.4, *p* = 0.009). As depicted in Figure 2A, UWT + R rats spent more time wall climbing (*n* = 9; Mean 4.71 ± 1.26 s) in comparison to Swim + R rats (*n* = 9; Mean 0.37 ± 0.21 s), indicating a difference in pattern of behavior during the test.

### Extracellular signal-regulated kinase (ERK2) activation in the hippocampus and the basolateral amygdala (BLA)

ERK2 activation (p-ERK2 as a percentage of total ERK2 expression) was measured 30 min after the exposure to the reminder and was normalized to the Naïve group values.

In the hippocampus, one-way ANOVA for ERK2 activation levels 30 min after the exposure to the reminder revealed a significant effect for group only in the VH. ERK2 activation in the dorsal hippocampal sub-regions did not differ significantly between the groups in the CA1 (*F*_(4,59)_ = 1.73, *p* = 0.157; Figure 1A) nor in the DG (*F*_(4,59)_ = 0.48, *p* = 0.749; Figure 1B). Within the VH, significant differences in ERK2 activation were found between the groups both in the CA1 (*F*_(4,59)_ = 5.43, *p* = 0.001)) and in the DG (*F*_(4,59)_ = 5.17, *p* = 0.001). *Post-hoc* LSD testing further showed that ERK2 activation in the UWT + R group was significantly higher than the rest of the groups both in the ventral CA1 (*p* < 0.01; Figure 1C) and the ventral DG (*p* < 0.01; Figure 1D).

One-way ANOVA for ERK2 activation levels in the BLA 30 min after the exposure to the reminder revealed a significant effect for group (*F*_(4,59)_ = 4.93, *p* = 0.001). *Post-hoc* LSD testing further showed that ERK2 activation in the UWT + R group was significantly higher than in the rest of the groups (*p* < 0.01; Figure 1E).

To further explore the relationship between specific region activation and the expression of stressful behavior during the reminder, we examined the correlation between time spent wall climbing during the reminder exposure and ERK2 activation in the different brain regions. A significant correlation was found between stressful behavior expression during the reminder and ERK2 activation only in the BLA (*r* = 0.77, *p* = 0.016) among rats in the UWT + R group. This correlation was not observed in the Swim + R group (Figure 2B).

The strong correlation between stressful behavior and ERK2 activation in the BLA is predicted by many models of fear memory retrieval (LeDoux, 2000, 2003; Zald, 2003; McGaugh, 2004). Different neural models in the past have suggested the involvement of both the hippocampus and BLA in stressful memory retrieval (Richter-Levin, 2004) while others also emphasizing the involvement of ventral regions of the hippocampus (Segal et al., 2010; Goosens, 2011). Therefore, we assessed potential correlations between ERK2 activation in the BLA and ERK2 activation in the hippocampus of all groups (summarized in Table 1). BLA ERK2 activation significantly correlated with Hippocampal ERK2 activation only in the ventral DG of UWT + R group (*r* = 0.67, *p* = 0.010; Figure 2C). No significant correlations were found between ERK2 activation in the BLA and ERK2 activation in the dorsal CA1 (Figure 2D), dorsal DG (Figure 2E) and ventral CA1 (Figure 2F) of animals exposed to the reminder.

**Table 1:** **Pearson correlations for ERK2 activation in the BLA and hippocampus**.

		**Dorsal CA1**		**Dorsal DG**		**Ventral CA1**		**Ventral DG**	
		**r**	**Sig.**	**r**	**Sig.**	**r**	**Sig.**	**r**	**Sig.**
Naive	**BLA**	.178	.581	.083	.797	−.068	.833	.389	.138
Swim	**BLA**	.469	.065	.253	.427	.124	.700	.412	.054
Swim+R	**BLA**	.150	.659	.177	.731	.415	.205	.126	.713
UWT	**BLA**	.126	.697	−.450	.142	−.506	.093	.079	.808
UWT+R	**BLA**	−.073	.821	−.020	.951	−.483	.112	.667	.010

## Discussion

Previous studies have demonstrated that exposure to stress results in increased activation of the amygdala (Roozendaal et al., 2009), and that stress-induced activation of the amygdala is likely to mediate memory-related processes in other brain areas, including the hippocampus (Cahill and McGaugh, 1998; Schwabe et al., 2012). Different levels of stress are associated with different levels of activation of the amygdala (e.g., Kogan and Richter-Levin, 2008), which may then modulate such memory processes accordingly (Canli et al., 2000; Li and Richter-Levin, 2012; Vouimba and Richter-Levin, 2013). It has been suggested that different emotional and stressful conditions will be associated with different maps of activation and of co-activity of brain regions associated with emotional memory. This “dynamic routing hypothesis” suggests that under more stressful, or traumatic conditions, there will be a shift in dominance of involvement, from more dorsal to more ventral parts of the hippocampus, and that there will be increased synchronization of ventral hippocampus with the activity in the amygdala (more particularly, in the BLA) (Kogan and Richter-Levin, 2008; Segal et al., 2010).

In line with our hypothesis, rats that were exposed to the reminder 24 h after exposure to a traumatic experience (UWT + R) exhibited higher levels of ERK2 activation in the ventral regions of the hippocampus and in the BLA. The increased levels of ERK2 activation in the BLA after an exposure to contextual reminder of fear memory have been shown in the past (Ilin and Richter-Levin, 2009; Ardi et al., 2013). In the hippocampus, UWT + R rats exhibited higher levels of ERK2 activation in the ventral CA1 and DG. Furthermore, the activation of their ventral DG positively correlated with their BLA activation which positively correlated with stressful behavior during the reminder exposure. This correlation may suggest a network interaction of the ventral DG with the BLA to regulate the expression of stressful behavior during exposure to reminder of a stressful experience.

ERK2 activation has been associated with neural plasticity and memory (Sweatt, 2001). The requirement of ERK2 activation in the hippocampus for the expression of LTM, induced by a fear conditioning paradigm, has been demonstrated before (Atkins et al., 1998). Moreover, the induction of hippocampal long-term potentiation (LTP) has been shown to depend on ERK2 activation (Adams and Sweatt, 2002). Stress is well known to modulate hippocampal LTP as well as hippocampus-dependent learning and memory. At the cellular level, elevated stress impairs LTP in dorsal CA1 (Korz and Frey, 2003; Howland and Wang, 2008), with complex effects on dorsal DG LTP (Bergado et al., 2011). Most of these studies have been conducted only in dorsal regions of the hippocampus (Howland and Wang, 2008). When tested directly, stress was found to have different effects on dorsal and VH LTP (Maggio and Segal, 2007, 2009). Together with these findings, our findings of a different pattern of ERK2 activation in the VH and its correlation to BLA activation in the aftermath of an exposure to a reminder of a traumatic event implies a pivotal role of VH activation under stressful conditions, as was hypothesized before (Segal et al., 2010).

The fact that ERK2, a molecular marker associated with plasticity, was found here to be differentially activated in the VH and BLA suggests that the exposure to the reminder cue of the trauma inflicts on memory-related mechanisms in a way that may lead to long-term changes. However, PTSD is a disorder that is characterized by lingering symptoms, lasting in humans more than 1 month after the trauma. Measured 24 h after the exposure to the traumatic experience, the current findings can relate to relatively early stages of traumatic memory consolidation. Co-activation among regions involved in the recall and potentially reconsolidation of traumatic experiences at such early stages might therefore represent an adaptive response. Never the less, co-activation among regions involved in the recall of traumatic memories was suggested to be an important mechanism in the particular way these memories are experienced by PTSD patients (Gilboa et al., 2004; Lanius et al., 2004). Understanding the neural mechanisms underlying these initial stages of traumatic memories formation may contribute to developing intervention strategies that could reduce the risk of eventually developing PTSD.

## Conflict of interest statement

The authors declare that the research was conducted in the absence of any commercial or financial relationships that could be construed as a potential conflict of interest.
